# Transcript Quantification by RNA-Seq Reveals Differentially Expressed Genes in the Red and Yellow Fruits of *Fragaria vesca*

**DOI:** 10.1371/journal.pone.0144356

**Published:** 2015-12-04

**Authors:** Yuchao Zhang, Weijia Li, Yujuan Dou, Junxiang Zhang, Guihua Jiang, Lixiang Miao, Guofen Han, Yuexue Liu, He Li, Zhihong Zhang

**Affiliations:** 1 College of Horticulture, Shenyang Agricultural University, 120 Dongling Road, Shenyang, 110866, China; 2 Institute of Horticulture, Zhejiang Academy of Agricultural Sciences, 139 Shiqiao Road, Hangzhou 310021, China; 3 Institute of Soil and Water Conservation of Liaoning Province, Chaoyang 122000, China; Zhejiang University, CHINA

## Abstract

*Fragaria vesca* (2*n* = 2*x* = 14), the woodland strawberry, is a perennial herbaceous plant with a small sequenced genome (240 Mb). It is commonly used as a genetic model plant for the *Fragaria* genus and the Rosaceae family. Fruit skin color is one of the most important traits for both the commercial and esthetic value of strawberry. Anthocyanins are the most prominent pigments in strawberry that bring red, pink, white, and yellow hues to the fruits in which they accumulate. In this study, we conducted a *de novo* assembly of the fruit transcriptome of woodland strawberry and compared the gene expression profiles with yellow (Yellow Wonder, YW) and red (Ruegen, RG) fruits. *De novo* assembly yielded 75,426 unigenes, 21.3% of which were longer than 1,000 bp. Among the high-quality unique sequences, 45,387 (60.2%) had at least one significant match to an existing gene model. A total of 595 genes, representing 0.79% of total unigenes, were differentially expressed in YW and RG. Among them, 224 genes were up-regulated and 371 genes were down-regulated in the fruit of YW. Particularly, some flavonoid biosynthetic pathway genes, including C4H, CHS, CHI, F3H, DFR and ANS, as well as some transcription factors (TFs), including MYB (putative MYB86 and MYB39), WDR and MADS, were down-regulated in YW fruit, concurrent with a reduction in anthocyanin accumulation in the yellow pigment phenotype, whereas a putative transcription repressor MYB1R was up-regulated in YW fruit. The altered expression levels of the genes encoding flavonoid biosynthetic enzymes and TFs were confirmed by quantitative RT-PCR. Our study provides important insights into the molecular mechanisms underlying the yellow pigment phenotype in *F*. *vesca*.

## Introduction

**Fig 1:**
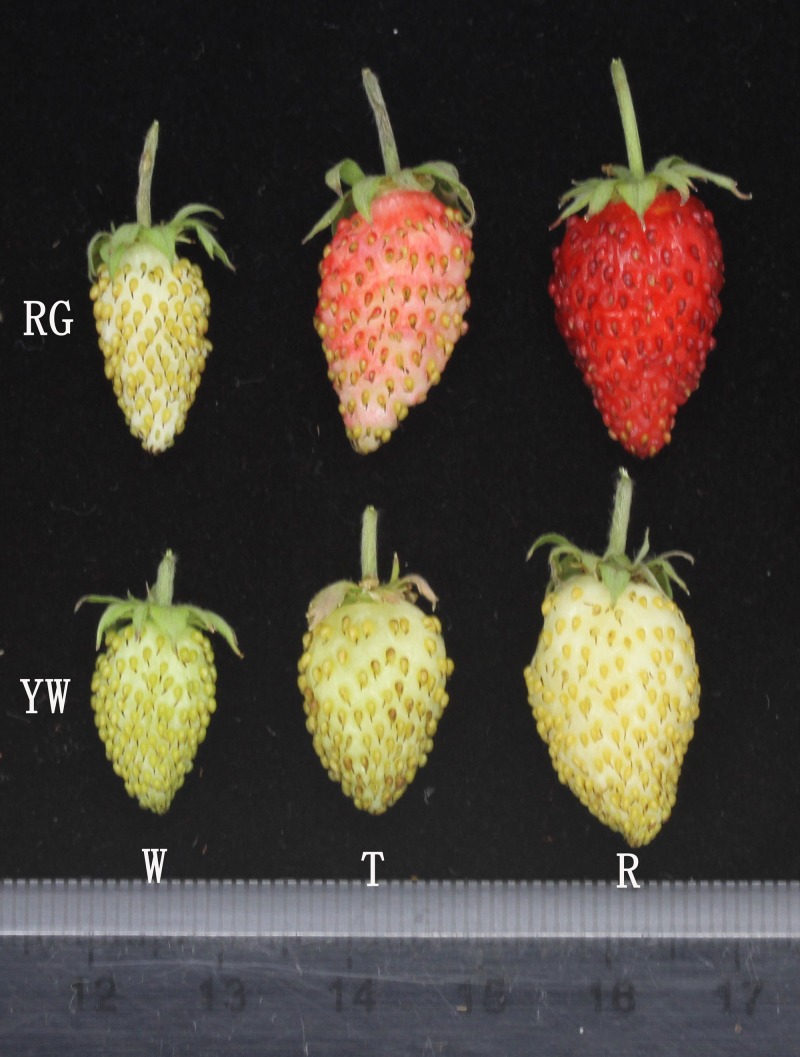
Developmental and ripening stages of YW and RG as defined in this research. Above: RG with white fruit, turning stage fruit and ripe fruit; Below: YW with white fruit, turning stage fruit and ripe fruit.

*Fragaria vesca*, commonly called woodland strawberry, is emerging as an advantageous alternative system for the cultivated octoploids as well as the Rosaceae family due to its small genome size (240 Mb), diploidy (2*n* = 2*x* = 14), small herbaceous stature, ease of propagation, short reproductive cycle, and facile transformation [1]. Woodland strawberry fruits are strongly flavored and have a wide variety of colors, such as red, yellow, white, and pink. Ruegen (RG) and Yellow Wonder (YW) are two botanical forms of *F*. *vesca*, both of which produce small-sized plants and propagate without runners. RG has fruits with red flesh and red skin, whereas YW fruits have both yellow flesh and skin (Fig 1). The availability of the *F*. *vesca* genomics resource affords opportunities to conduct comparative gene studies within the Rosaceae and identify important genes involved in flavonoid biosynthesis in strawberry [1].

Anthocyanins are widely distributed in seed plants and responsible for orange to blue colors in various tissues, such as flowers, fruits, leaves, and seeds [2]. Numerous publications have confirmed that anthocyanins are derived from a plant secondary metabolite pathway, known as the flavonoid biosynthetic pathway [3]. The flavonoid biosynthetic pathway has been extensively studied in a number of plant species, and was recently described in strawberry [4,5]. Fruit pigmentation in strawberry appears to be determined by the expression of a set of genes involved in flavonoid biosynthetic pathway, including C4H (cinnamate 4-hydroxylase), CHS (chalcone synthase), CHI (chalcone isomerase), F3H (flavanone 3-hydroxylase), F3′H (flavonoid 3′-hydroxylase), DFR (dihydroflavonol-4-reductase), ANS (anthocyanidin synthase) and 3-GT (3-glycosyltransferase) [6,7], which are coordinated by regulatory proteins called transcription factors (TFs), such as MYB, bHLH, MADS, and WRKY [8,9].

RNA-Seq is a powerful, accurate and cost-effective method that produces millions of short cDNA reads [10]. The reads are aligned to both reference-based transcriptome assembly and *de novo* transcriptome assembly, to produce a genome-scale transcriptional profile for investigating transcriptional regulation [11]. RNA-Seq has been applied successfully in transcriptome profiling of species without genome sequencing data [12].

In this paper, we present a *de novo* assembly of the fruit transcriptome of *F*. *vesca* using Illumina-based RNA-Seq data. Differential gene expression between the red fruit and the yellow fruit was investigated to reveal the differential regulation of key pathways.

## Materials and Methods

### Plant material

*Fragaria vesca* accessions (Ruegen, RG) and (Yellow Wonder, YW) were grown in pots and maintained at Shenyang Agricultural University. Ruegen (*F*. *vesca* f. semperflorens D), the first modern cultivar, i.e., runnerless, everbearing and red fruited, originated from Castle Putbus in Germany. Yellow Wonder (*F*. *vesca* f. alba E) was first found in California, USA. YW has the recessive mutant traits, yellow fruit and runnerless. Three development and ripening stages were distinguished based on the weight and color of the receptacle: W, white fruit; T, turning stage; R, ripe fruit. Two biological replicates of each fruit sample were collected and immediately stored at -80°C after being quickly frozen in liquid nitrogen until use.

### RNA extraction and quality assessment

Total RNA were isolated using the modified CTAB method described by Chang et al. [13], and the RNA samples were treated with DNase (TaKaRa, Japan) for 4 h. The integrity of the RNA samples was examined using an Agilent 2100 Bioanalyzer (Agilent Technologies, Palo Alto, USA).

### cDNA library preparation and Illumina sequencing

Total RNA samples of RG and YW fruits at turning stage in two biological replicates were submitted to Biomarker Technology Company, Beijing, China for cDNA library preparation and sequencing reactions. The paired-end library preparation and sequencing were performed following standard Illumina methods using a DNA sample kit (#FC-102-1002, Illumina). The cDNA library was sequenced on the Illumina sequencing platform (HiSeq^™^ 2500).

### *De novo* assembly

Reads from each library were assembled separately. The trimming adapter sequences were removed and low-quality reads (with unknown nucleotides larger than 5%) were filtered by the Biomarker Technology Company. The Trinity method [14] was used for *de novo* assembly of Illumina reads of woodland strawberry. Trinity consisted of three software modules—Inchworm, Chrysalis and Butterfly—applied sequentially to process large volumes of RNA-Seq reads. In the first step in Trinity, reads were assembled into the contigs by the Inchworm program. The minimally overlapping contigs were clustered into sets of connected components by the Chrysalis program, and then the transcripts were constructed by the Butterfly program [14]. In this study, only one k-mer length (25-mer) was chosen in Trinity, using the follow parameters: seqType fq, group pairs distance = 150 and other default parameters. Finally, the transcripts were clustered by similarity of correct match length beyond 80% of the longest transcript or 90% of the shortest transcript used multiple sequence alignment tool BLAT [15]. The longest transcript of each cluster was taken as the unigene. The Illumina data set has been deposited in the NCBI Sequence Read Archive (SRA) under accession number SRX1294640.

### Functional annotation

We annotated unigenes based on a set of sequential BLAST searches [16] to find the most descriptive annotation for each sequence. The assembled unigenes were compared with sequences in the National Center for Biotechnology Information (NCBI) non-redundant (Nr) protein and nucleotide (Nt) databases (http://www.ncbi.nlm.nih.gov), the Swiss-Prot protein database (http://www.expasy.ch/sprot), the Kyoto Encyclopedia of Genes and Genomes (KEGG) pathway database (http://www.genome.jp/kegg), the Cluster of Orthologous Groups (COG) database (http://www.ncbi.nlm.nih.gov/COG), the Translated EMBL Nucleotide Sequence database (TrEMBL) (http://www.uniprot.org/), and the Protein family (Pfam) database (http://pfam.xfam.org/). The Blast2GO program [17] was used to obtain GO annotation of the unigenes. WEGO software (http://wego.genomics.org.cn/cgi-bin/wego/index.pl) was then used to perform GO functional classification of all unigenes to view the distribution of gene functions.

### Digital gene expression analysis

Gene expression levels were measured in the RNA-Seq analysis as reads per kilobase of exon model per million mapped reads (RPKM) [18]. DESeq software [19] was used to identify differentially expressed genes (DEGs) in pair-wise comparisons, and the results of all statistical tests were revised for multiple testing with the Benjamini—Hochberg false discovery rate (FDR < 0.01). Sequences were deemed to be significantly differentially expressed if the adjusted *P* value obtained by this method was <0.001, and there was at least a twofold change (>1 or <− 1 in log 2 ratio value) in RPKM between the two libraries.

### Quantitative RT-PCR (qRT-PCR) analysis

cDNA was synthesized using Reverse Transcriptase XL (AMV) (TaKaRa, Japan) according to the manufacturer’s protocol in a 20 μL reaction system. The reverse transcription reaction mixture contained 5 μL total RNA (1 μg), 1 μL of each 10 mM dNTPs, 1 μL of random primer (9 mer) (50 μM), 1 μL oligo d(T)_18_ primer (50 μM) (TaKaRa, Japan), and 6 μL DEPC water. The mixture were incubated at 65°C for 5 min and cooled on ice for 5 min, then 4 μL 5× Reverse Transcriptase buffer, 1 μL RNasin (TaKaRa, Japan), and 1 μL AMV (5 U) were added. The mixture was incubated at 37°C for 2.5 h, and then the enzyme was inactivated by incubating at 72°C for 15 min. qPCR was carried out in an iQ5 Real Time PCR Detection System (BioRad, USA) with RealMasterMix SYBR Green (TIANGEN, China). Primers used for validation of differentially expressed genes are shown in S1 Table. All data were normalized with the level of the *Fv26S* internal transcript control. Relative fold changes in genes expression were calculated using the comparative Ct (2^-ΔCt^) method. Each sample was quantified in triplicate.

### Total flavonoid content analysis

Total flavonoid analysis of fruit extracts in methanol was carried out based on the method of Swain and Hillis [20]. The flavonoid content was measured using a colorimetric assay developed by Jia et al. [21]. Absorbance was read at 510 nm against the blank (water) and flavonoid content was expressed as mg per gram of fresh weight.

### Total anthocyanin content analysis

Determination of total anthocyanin content was performed following the methods detailed previously by Pirie and Mullins [22]. Total anthocyanin content was expressed as nmol per gram of fresh weight.

## Results

### Quantification of anthocyanins and flavone in YW and RG

**Fig 2:**
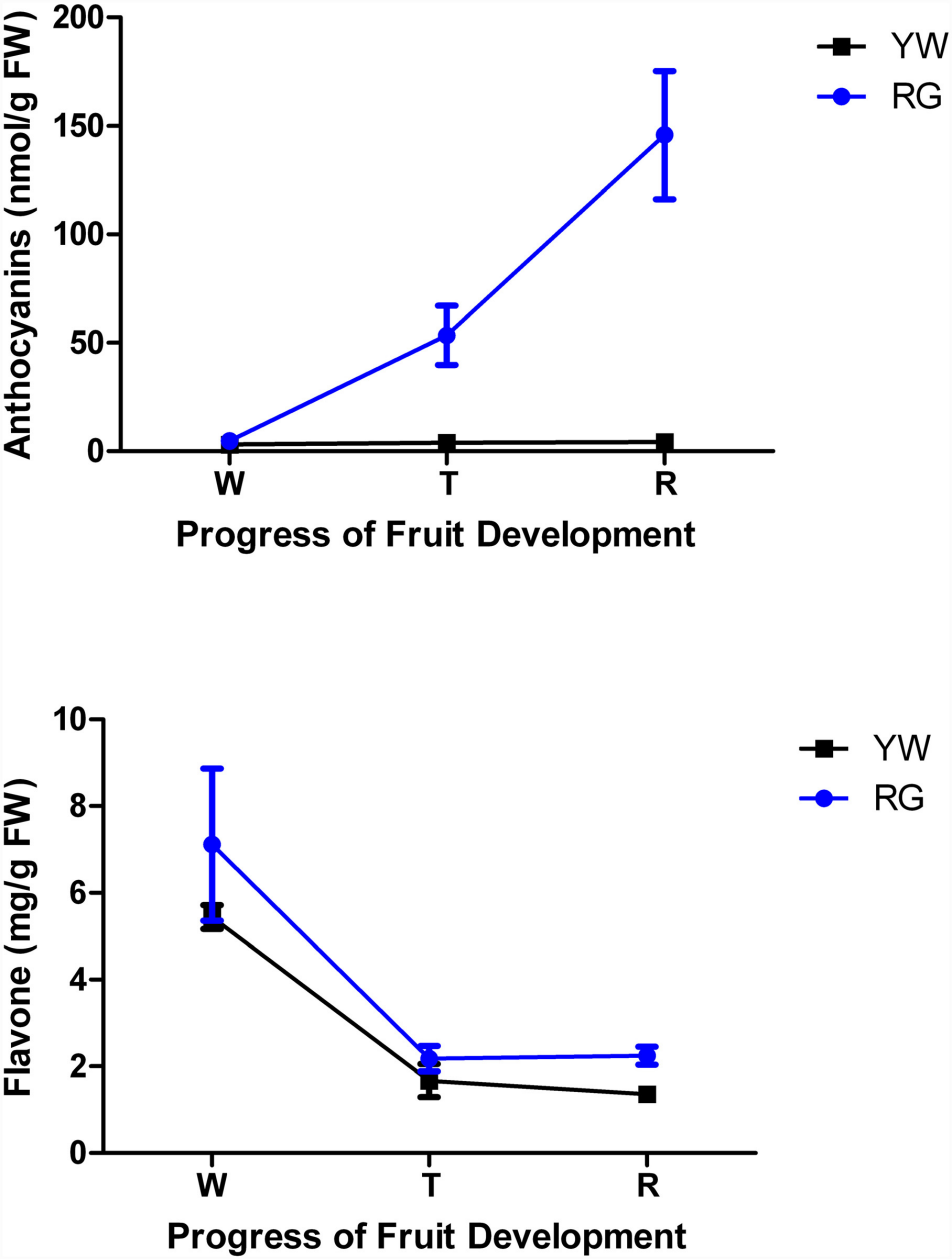
Quantification of anthocyanins and flavone in YW and RG. Blue lines represent Ruegen (RG), black lines represent Yellow Wonder (YW). The Y-axis represents the content of anthocyanins and flavone; the X-axis represents the progress of fruit development. W indicates white fruit; T indicates turning stage; R indicates ripe fruit.

The anthocyanins and total flavone in YW and RG (Fig 1), two botanical forms of *F*. *vesca* with significant difference in fruit pigmentation, were examined. RG fruit, showed a continually increase in anthocyanins concomitant with the progress of fruit development and ripening. Conversely, no significant variation in the amounts of anthocyanins in YW fruit was observed during fruit ripening progress (Fig 1). High levels of anthocyanins were detected in red fruit from the turning stage to ripening, but not in yellow fruit. This difference led to the different phenotypic characteristics of the two types of *F*. *vesca* fruit. However, the flavone concentration in YW is not affected by the low levels of anthocyanins. YW and RG showed a similar trend in flavone accumulation. The amounts of flavone were highest in immature stages, and decreased concomitant with fruit ripening, reached a similar level in YW and RG at the ripe fruit stage. The amount of flavone in RG was slightly higher than that in YW when the fruit was mature (Fig 2).

### *De novo* assembly and assessment of the Illumina ESTs

For RNA-Seq analysis, a total of four cDNA libraries were prepared using material from the turning stage fruits of RG and YW in two biological replicates. After removing low-quality reads and trimming adapter sequences, approximately 81.4 million high-quality pair-end reads from RG and YW were obtained, encompassing over 20.5 billion nucleotides (nt) of sequence data (Table 1). *De novo* assembly was carried out using the software Trinity to construct the full-length transcript, which was designed specifically for high-throughput RNA sequencing [23]. The mean length of contigs was approximately 42 bp, and the number of >200 bp contigs was 84,939 (Table 1). The transcripts were constructed using the Butterfly program of Trinity. Total of 139,997 transcripts were obtained, with average lengths of approximately 1,295 bp (Table 1).

**Table 1:** Summary of RNA-Seq and *de novo* assembly of *Fragaria vesca* unigenes.

Sequences	
Total nucleotides	20,501,684,236
Number of clean reads	81,421,741
Number of >200-bp contigs	84,939
Mean length of contigs (bp)	42.43
N50 length of contigs (bp)	43
Number of >200-bp transcripts	139,997
Mean length of transcripts (bp)	1,294.78
N50 length of transcripts (bp)	2,272
Number of Unigenes	75,426
Mean length of Unigenes (bp)	750.01
N50 length of Unigenes (bp)	1,380

These transcripts were assembled into unigenes. After combining the unigene data from RG and YW, a unigene database for strawberry containing 75,426 unigenes was established. The total length of the unigenes was 56,570,128 bp, and the mean length of individual unigenes was 750 bp. Among all the strawberry unigenes, 16,034 have lengths of more than 1,000 bp, representing 21.3% (16,034/75,426) of the total unigenes (Table 2). The size distribution of the assembled unigenes is shown in S1 Fig.

**Table 2:** The size distribution of *Fragaria vesca* unigenes.

Length of unigene (bp)	No. of unigenes	Percentage (%)
200–300	27,798	36.85
300–500	18,384	24.37
500–1,000	13,210	17.51
1,000–2,000	9,486	12.58
2000+	6,548	8.68
Total	75,426	

### Functional annotation and characterization of unigenes

**Fig 3:**
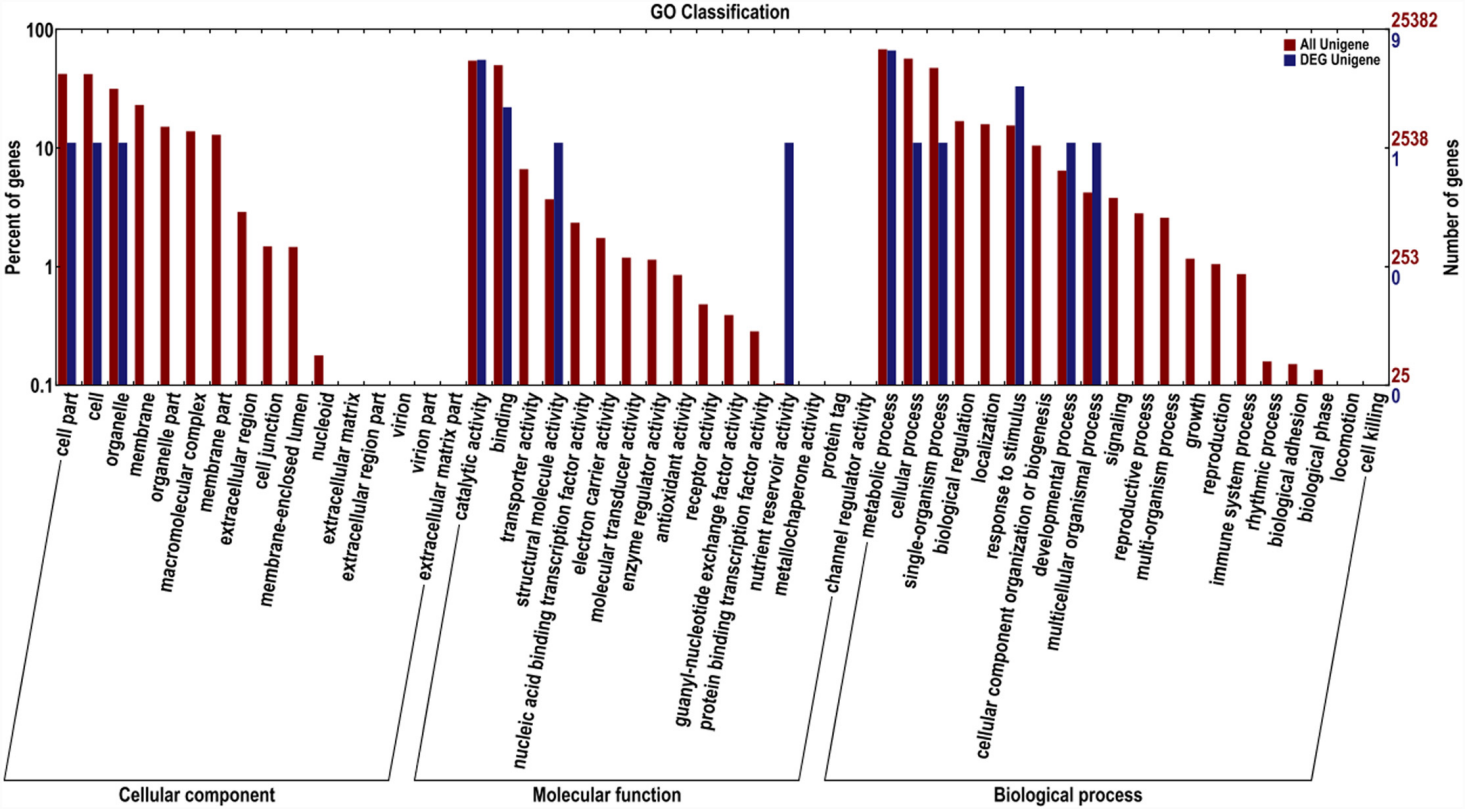
Histogram of the GO classifications of assembled *Fragaria vesca* unigenes. The results are summarized in three main GO categories: cellular component, molecular function, and biological process.

The entire unigenes were annotated on the basis of similarities to known or putative sequences in the web databases. Among the 75,426 unique sequences, 45,387 (60.2%) had at least one significant match to an existing gene model in BLAST searches (Table 3). Based on sequence homology, the unigenes of *F*. *vesca* were categorized into 52 functional groups, belonging to three main GO ontologies: cellular component, molecular function, and biological process (Fig 3). The results showed a high percentage of genes from the categories “metabolic process”, “cellular process”, “catalytic activity”, “binding” and “single-organism process”, with only a few genes related to “channel regulator activity”, “cell killing”, and “protein tag”.

**Table 3:** Summary of annotations of assembled strawberry (*Fragaria vesca*) unigenes.

Category	Number of unigenes ≥300 bp	Number of unigenes ≥1,000 bp	Total number of annotated unigenes	Percentage (%)^b^
COG_Annotation	11,584	6,617	14,710	19.5
GO_Annotation	17,979	8,847	25,382	33.7
KEGG_Annotation	7,568	3,923	10,067	13.3
KOG_Annotation	17,925	9,332	23,400	31.0
Swiss-Prot_Annotation	20,933	11,770	26,066	34.6
Pfam_Annotation	21,802	12,800	26,539	35.2
TrEMBL_Annotation	27,790	14,612	27,790	36.8
Nr^a^_Annotation	32,047	14,904	45,191	59.9
All_Annotation	32,136	14,915	45,387	60.2

^a^ Nr = NCBI non-redundant sequence database

^b^ Proportion of the 75,426 assembled unigenes

### Transcript differences between RG and YW

**Fig 4:**
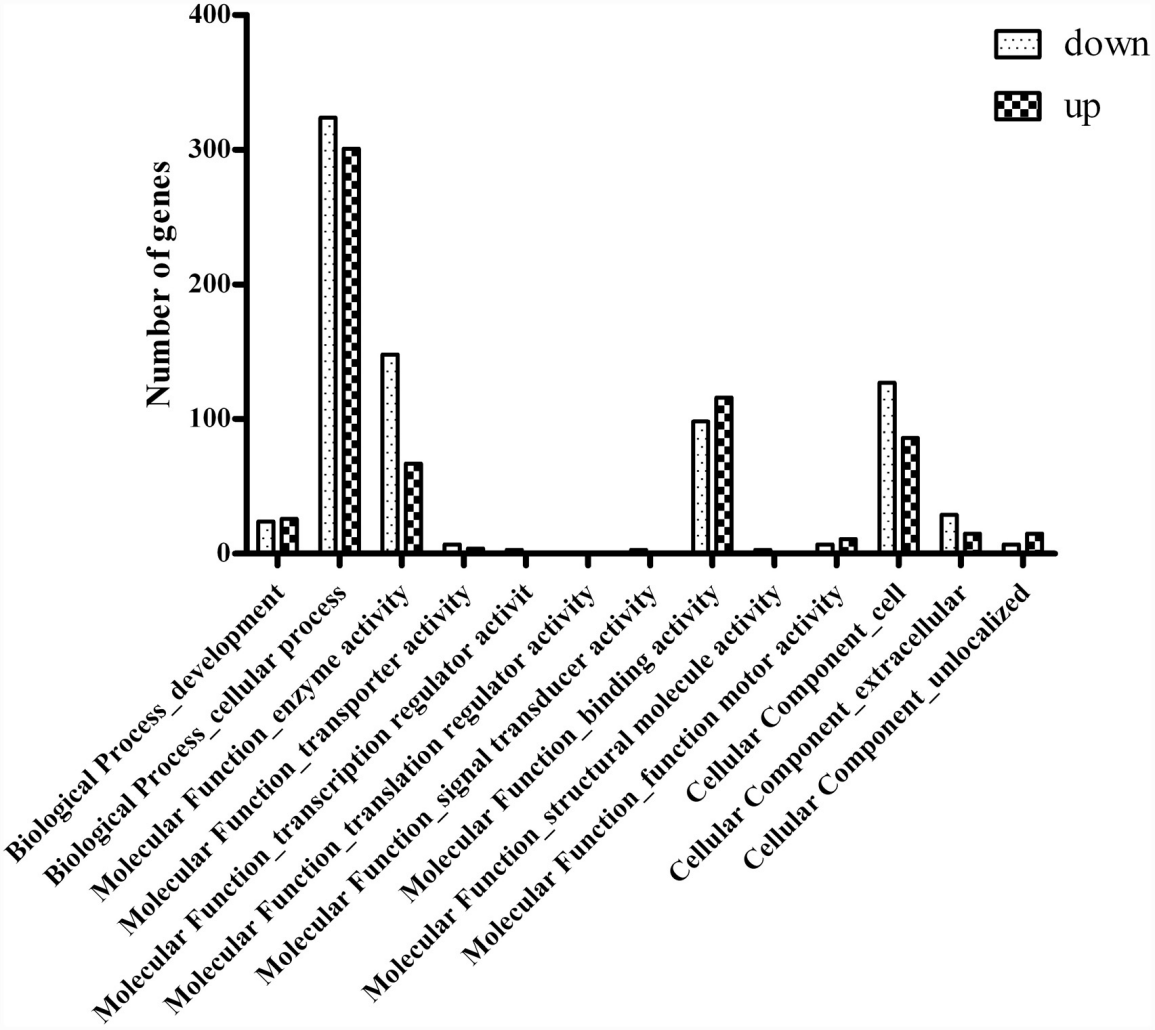
Functional categories of 595 unigenes differentially expressed in *Fragaria vesca* accessions Yellow Wonder (YW) and Ruegen (RG). Dark-colored bars indicate the genes up-regulated and light-colored bars indicate the genes down-regulated.

The general chi-squared test was used with a random sampling model in the DESeq software [19] to identify DEG in the turning stage fruits of RG and YW. A total of 595 genes, representing 0.79% (595/75,426) of the total unigenes, were differentially expressed in RG and YW (twofold or more change; *p* < 0.001) in both biological replicates. The detailed information on these genes is presented in S2 Table. Among the DEGs in the two types of fruit, 224 genes were up-regulated and 371 genes were down-regulated in YW. In addition, 98.0% (583/595) of the DEGs were detected in the fruits of both accessions. GO functional classes were assigned to the DEGs with putative functions. These genes were sorted into major functional categories (Fig 4). In an effort to identify key genes responsible for anthocyanin deposition in the fruit skin, flavonoid biosynthetic pathway genes were identified from the 595 DEGs.

Some flavonoid pathway genes were down-regulated in the fruits of YW compared with the fruits of RG in both biological replicates, including the genes encoding C4H [EC 1.14.13.11], CHS [EC 2.3.1.74], CHI [EC 5.5.1.6], F3H [EC 1.14.11.9], DFR [EC 1.1.1.219] and ANS [EC 1.14.11.19] (Table 4). The gene expression ratios for each selected flavonoid pathway gene were shown in the flavonoid biosynthetic pathway [KEGG map00941 (http://www.genome.jp/kegg-bin/show_pathway?map00941)], and detailed information is presented in S1 File.

**Table 4:** Expression profiles of flavonoid biosynthesis genes in *Fragaria vesca*.

Annotation	Unigene ID	Function	log_2_(YW/RG)
C4H	c19945.graph_c0	Flavonoid biosynthetic	-1.78
CHS (CHS2/CHS5)	c7666.graph_c0	Flavonoid biosynthetic	-1.42
CHI	c24522.graph_c0	Flavonoid biosynthetic	-1.64
F3H	c24499.graph_c0	Flavonoid biosynthetic	-1.29
DFR (DFR1)	c24714.graph_c0	Flavonoid biosynthetic	-1.19
ANS	c24538.graph_c0	Flavonoid biosynthetic	-2.35
MYB1	c32867.graph_c0	Transcription factor	-4.36
MYB1R (MYB1R1)	c16952.graph_c0	Transcription factor	3.95
MYB86-like	c10204.graph_c0	Transcription factor	-1.80
MYB39-like	c18291.graph_c0	Transcription factor	-1.84
WD-repeat (WDR)	c19202.graph_c0	Transcription factor	-2.03
MADS (AGL11-like)	c14603.graph_c0	Transcription factor	-2.26
MADS (AGL15-like)	c20999.graph_c0	Transcription factor	-1.95

To identify regulatory factors that potentially controlled flavonoid biosynthesis, candidate transcription factors were chosen from the transcriptome data. We initially focused on MWB (MYB-bHLH-WD40) complex proteins, which were key factors in the regulation of primary and secondary metabolism [24]. Interestingly, one candidate MYB gene (c32867.graph_c0) annotated as the MYB1 gene that was known to control anthocyanin biosynthesis was identified [25], which was strongly down-regulated in the yellow fruit (YW) compared with the red fruit (RG) [log_2_(YW/RG) = -4.36] in both biological replicates. Some other TFs were down-regulated in YW, including two putative MYB TFs (annotated as MYB86 and MYB39), two putative MADS TFs (annotated as AGL11-like and AGL15-like gene), one putative WD-repeat protein (WDR) TFs (Table 4). Noticeably, a single-repeat MYB genes (Table 4), annotated as MYB1R and previously known as a repressors of anthocyanin accumulation [26], was significantly up-regulated in YW [log_2_ (YW/RG) = 3.95].

### Quantitative RT-PCR (qRT-PCR) analysis of the flavonoid pathway genes and TFs

**Fig 5:**
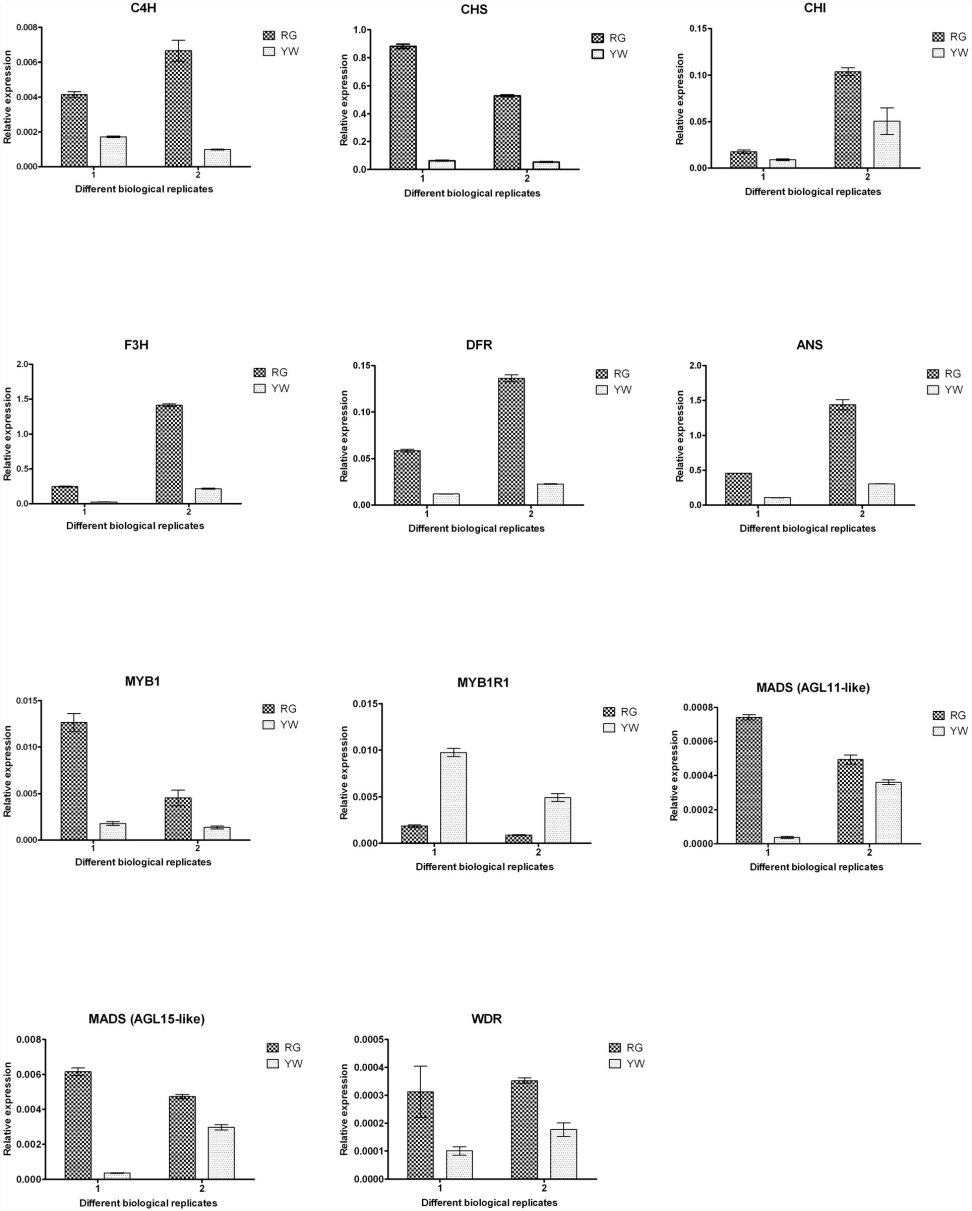
The expression levels of six flavonoid pathway genes and the TFs at developmental stage T between two biological replicates samples using qRT-PCR. Dark-colored bars indicate Ruegen (RG), light-colored bars indicate Yellow Wonder (YW). The Y-axis represents relative expression; the X-axis represents the different biological replicates.

To confirm the results of the Illumina RNA-Seq analysis, the expression levels of six flavonoid pathway genes (C4H, CHS, CHI, F3H, DFR and ANS) and the differently expressed TFs (MYB1, MYB1R, WDR, MADS) were tested in RG and YW by qRT-PCR using the same samples prepared for RNA-Seq. According to the qRT-PCR data (Fig 5), the transcript levels of the six flavonoid pathway genes and the TFs (MYB1, WDR, MADS) were greatly reduce in YW compared with RG, whereas MYB1R was clearly up-regulated in the fruits of YW with respect to RG. As shown in Fig 5, all chosen genes examined by qRT-PCR showed the same trends of mRNA accumulation patterns as identified in the RNA-Seq data.

## Discussion

The genome of *F*. *vesca* was sequenced in 2010. This provided an invaluable resource for studying the molecular mechanisms influencing strawberry development. When a reference genome is available, the sequencing reads are aligned primarily by mapping on to the sequenced reference genome. However, although reference-based approaches is a robust and relatively precise way of characterizing transcript sequences, this method remains problems by its inability to account for un-sequenced genome or structural alterations within mRNAs, such as spliced variants, and also does not solve the problem of hypervariable sequences and private genes [23,27,28]. Moreover, relying on a single reference genome may underrate the variability among different genotypes. These challenges can be addressed by using a *de novo* assembly strategy. *De novo* assembly can reconstruct short sequences of transcripts into entire sequences of transcriptomes, identify all of the expressed genes, separate isoforms and quantify transcript expression levels, which do not depend on the genome [29]. In this study, a total of 20.5 Gb of raw sequence data were generated by Illumina sequencing of two botanical types of *F*. *vesca*, RG and YW, corresponding to 75,426 unigenes.

Genome-wide transcriptome analysis of the red and yellow strawberry fruit using RNA-Seq technology revealed significant down-regulation of a number of flavonoid pathway genes, both early biosynthetic genes C4H, CHS as well as CHI and late biosynthetic genes ANS and DFR (Table 4) concurrent with a reduction in anthocyanins accumulation and yellow pigment phenotype. Previous researches indicated that the lack of anthocyanin pigmentation seems to be caused by the down-regulation of these flavonoid pathway genes, i.e., the expression levels of CHS, F3H, DFR, ANS were less pronounced in yellow apple cultivar ‘Orin’ compared to the red apple cultivar ‘Jonathan’ [30]. The lack of anthocyanins in the Caryophyllales is caused by the suppression or limited expression of the DFR and ANS [31]. And, the lack of color in white native Chilean strawberry may be also attributed to the low expression of ANS [32]. Moreover, a candidate gene approach was used to determine the likely molecular identity of the *c* locus (yellow fruit color) in *F*. *vesca*, and the results showed that the *c* locus were tightly linked with the F3H gene [33], which suggested that F3H was necessary for red fruit color in *F*. *vesca*. RNAi silencing of F3H in strawberry fruits also exhibited that the anthocyanin content was greatly reduced and flavonol was also decreased [6]. Recently, it was found that differing hydroxylation pattern of anthocyanins in *F*. *vesca* and *F*. ×*ananassa* was reflected in the expression of F3’H and DFR1, and F3’H deficient lines displayed white or pale pigmentation phenotype [34]. In this study, a set of flavonoid pathway genes were down-regulated in yellow woodland strawberry fruits, including C4H, CHS, CHI, F3H, DFR and ANS, which indicated the transcript abundance of these genes were positively related to the accumulation of anthocyanin. In addition, the expression level of F3’H mentioned above showed no significant difference between yellow and red fruits of *F*. *vesca* in our RNA-Seq data. The co-down-regulation of many structural genes in YW indicates that yellow fruit phenotype is unlikely a mutation of one specific flavonoid pathway gene and multiple genes or transcription factors interacting with each other may account for the yellow coloration of woodland strawberry fruit.

The structural genes of plant flavonoid biosynthetic pathway are largely regulated at transcriptional level. The R2R3-MYB TFs played a key role in the regulation of the flavonoid pathway in most plant species [35,36]. R2R3-MYB TFs can interact or not with bHLH proteins and/or with WDR proteins [37]. Two of them, known as MYB10 and MYB1, have been extensively studied in numerous plant species and were recently described in strawberry. MYB10 regulates the expression of most of the early biosynthetic genes and the late biosynthetic genes involved in anthocyanin production in ripened strawberry fruits [38]. Over-expression the *FvMYB10* in ‘Alpine’ strawberry *F*. *vesca* resulted in plants with elevated leaves, petioles, stigmas and fruit anthocyanin concentrations; while the mature fruit of *FvMYB10* RNAi lines showed white fruit skin and white flesh [39]. *FaMYB1* was described as a transcriptional repressor in regulating the biosynthesis of anthocyanins and flavonols in strawberry [25]. *FvbHLH33*, which is a potential bHLH partner for *FvMYB10*, did not affect the anthocyanin pathway when knocked down using an RNAi construct [39]. Suppressed expression of MBW complex protein encoded by *FaTTG1* gene caused enhanced anthocyanin accumulation in strawberry fruit off plant [40].

In this study, the MBW members reported in strawberry (*FvMYB10*, *FvbHLH33* and *FaTTG1*) were not involved in DEGs. Unexpectedly, MYB1 gene was significant down-regulated in the fruits of YW, and it was previous reported that the high level of transcripts of *FcMYB1* was detected in white Chilean strawberry [32]. One possible explanation is the expression levels of MYB1 may be not the main cause of the loss of anthocyanins in YW fruits. However, MYB10 was well-known as a major regulator of anthocyanin biosynthesis in fruits [8,38]. Thus, MYB10 was still considered as an important candidate gene for anthocyanin accumulation in strawberry in this study. The coding regions of *FvMYB10* from YW and RG were sequenced and a missense mutation was found in *FvMYB10* due to a G to C base substitution at the 35th nucleotide of the cDNA sequence of YW, resulting in an amino acid Trp to Ser change (S2 File). The missense mutation might result in *FvMYB10* gene dysfunction and affect many structural genes participated in flavonoid pathway biosynthesis, which might lead to the yellow color fruit phenotype in YW. Functional studies of the point mutation in *FvMYB10* will be the next step to gain a better understanding of pigment-deficient phenotype in YW fruits.

In addition, a number of other TFs were differentially expressed in RG and YW, including two putative MYB TFs (annotated as MYB86 and MYB39), two putative MADS TFs (annotated as AGL11-like and AGL15-like), one putative WDR TF and one R3 single-repeat MYB TF (annotated as MYB1R). Among these TFs, MYB86 and MYB39 have not been proved to be involved in flavonoid biosynthesis until now. WDR is more likely to enhance gene activation rather than a direct regulatory function because it commonly has no obvious catalytic activity [37,41]. MYB1R TFs were reported as transcription repressors of anthocyanin biosynthesis in tobacco, *Arabidopsis* and *Mimulus* [26,42,43]. Overexpression of two novel MYB1R TFs (*GtMYB1R1* and *GtMYB1R9*) in tobacco flowers induced a decrease in anthocyanin accumulation [26]. MADS is a highly conserved sequence motif found in a family of transcription factors. In plants, MADS genes commonly regulate the development of flower, ovule, fruit, leaf and root [44,45,46]. Recently, MADS genes were found in the plants that have been associated with flavonoid metabolism. *ABS/TT16* from *Arabidopsis* is a member of Bs MADS subfamily *GGM13*-like gene, which was initially found in the control of flavonoid biosynthesis in the yellow seed coat [47]. A new MADS gene (*IbMADS10*) from sweet potato (*Ipomoea batatas* L.) was reported to be related to the red pigmentation [48]. Over-expressing *IbMADS10* gene in *Arabidopsis* showed high accumulation of the anthocyanin pigments [49]. Silencing a *SQUAMOSA*-class MADS transcription factor, *VmTDR4*, resulted in substantial reduction in anthocyanin levels in ripe bilberry (*Vaccinium myrtillus*) fruits [50]. A gene named *PyMADS18* and putatively involved in anthocyanin biosynthesis was found in pear (*Pyrus communis* L.) [51]. Therefore, the limited expression of MADS TFs and/or the high levels of transcription repressors MYB1R may also contribute to the pigment-deficient phenotype of YW fruits.

In summary, our results showed the use of RNA-Seq technology to perform a *de novo* assembly of the fruit transcriptomes of two botanical forms of *F*. *vesca* contrasting in fruit pigmentation. Our data revealed significant down-regulation of certain flavonoid biosynthetic genes, including C4H, CHS, CHI, F3H, DFR and ANS, concomitant with the pigment-deficient phenotype. In addition, we have identified some transcription factors including MYB, WDR and MADS that potentially control flavonoid biosynthesis.

## Supporting Information

S1 Fig The size distribution of *Fragaria vesca* unigenes. (EPS)

S1 File Flavonoid biosynthetic pathway (KEGG map00941) genes differentially expressed in fruits of Yellow Wonder (YW) and Ruegen (RG). (RAR)

S2 File The cDNA sequence of *FvMYB10* gene in fruits of Yellow Wonder (YW) and Ruegen (RG). (DOC)

S1 Table Primers used to perform qPCR of flavonoid biosynthesis and regulatory genes. (XLS)

S2 Table The unigenes differentially expressed in Yellow Wonder (YW) and Ruegen (RG). (XLS)
