# Supplementary figures and images for: Transcript Quantification by RNA-Seq Reveals Differentially Expressed Genes in the Red and Yellow Fruits of Fragaria vesca
Source: PLoS One. 2015 Dec 4;10(12):e0144356. doi: 10.1371/journal.pone.0144356 (PMC4670188; doi:10.1371/journal.pone.0144356)

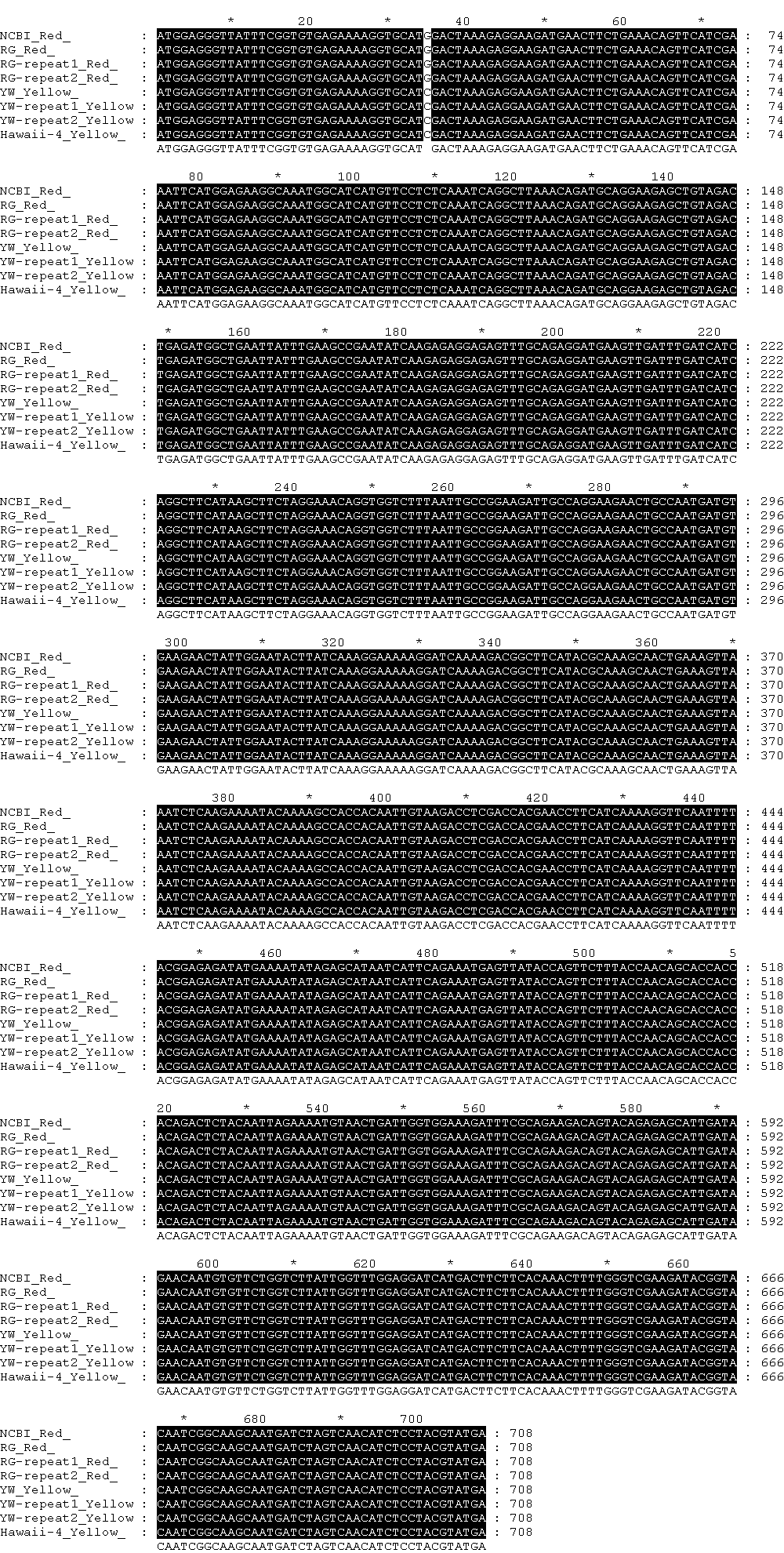

Supplement: S2 File — (DOC) [file pone.0144356.s003.doc]
